# Developing a Radiomics Atlas Dataset of normal Abdominal and Pelvic computed Tomography (RADAPT)

**DOI:** 10.1007/s10278-024-01028-7

**Published:** 2024-02-21

**Authors:** Elisavet Kapetanou, Stylianos Malamas, Dimitrios Leventis, Apostolos H. Karantanas, Michail E. Klontzas

**Affiliations:** 1 Biomedical Engineering Graduate Programme, School of Medicine, University of Crete, Heraklion, Greece; 2 Department of Computer Science-University of Crete, Heraklion, Greece; 3 Department of Medical Imaging, University Hospital of Heraklion, Crete, Greece; 4 Department of Radiology, School of Medicine, University of Crete, Heraklion, Greece; 5 Computational Biomedicine Laboratory, Institute of Computer Science, Foundation for Research and Technology (FORTH), Heraklion, Crete, Greece

**Keywords:** Radiomics, CT, Dataset, Αbdomen, Pelvis

## Abstract

Atlases of normal genomics, transcriptomics, proteomics, and metabolomics have been published in an attempt to understand the biological phenotype in health and disease and to set the basis of comprehensive comparative omics studies. No such atlas exists for radiomics data. The purpose of this study was to systematically create a radiomics dataset of normal abdominal and pelvic radiomics that can be used for model development and validation. Young adults without any previously known disease, aged > 17 and ≤ 36 years old, were retrospectively included. All patients had undergone CT scanning for emergency indications. In case abnormal findings were identified, the relevant anatomical structures were excluded. Deep learning was used to automatically segment the majority of visible anatomical structures with the TotalSegmentator model as applied in 3DSlicer. Radiomics features including first order, texture, wavelet, and Laplacian of Gaussian transformed features were extracted with PyRadiomics. A Github repository was created to host the resulting dataset. Radiomics data were extracted from a total of 531 patients with a mean age of 26.8 ± 5.19 years, including 250 female and 281 male patients. A maximum of 53 anatomical structures were segmented and used for subsequent radiomics data extraction. Radiomics features were derived from a total of 526 non-contrast and 400 contrast-enhanced (portal venous) series. The dataset is publicly available for model development and validation purposes.

**Supplementary Information:**

The online version contains supplementary material available at 10.1007/s10278-024-01028-7.

## Introduction

The advent of high-throughput technologies for molecular characterization has enabled the creation of tissue and organ atlases defined in human health and disease [1–4]. Genomic, transcriptomic, proteomic, and metabolomic characterization is becoming increasingly available in public repositories from large research initiatives and consortiums creating human health reference atlases [2–6]. The creation of open access reference databases with large-scale molecular characterization of normal organs, tissues, and cells is intended to set the basis for comparative studies, by providing open data to detect pathologies, study different developmental stages, and understand the mechanisms underlying biological functions in healthy and pathological states.

Despite the ever-growing availability of molecular characterization atlases of physiological organs with large-scale quantitative data, respective open access resources and databases for normal image-based “omics” (i.e., radiomics) remain extremely limited. Although public radiology imaging (i.e., MRI, CT, PET) and radiomics datasets are currently available, in most cases they are specific to a single organ or anatomic structure and mostly focused on diseased states, such as The Cancer Imaging Archive (TCIA) [7–10]. Respective open access resources and databases for biomedical imaging and normal image-based omics remain extremely limited and mostly focused on a single organ [11]. Publicly available datasets are crucial for validating and evaluating machine learning and imaging biomarker research for applications in radiology.

The aim of this work was to create the Radiomics Atlas Dataset of normal Abdominal and Pelvic computed Tomography (RADAPT), a publicly available radiomics dataset of 53 normal organs and anatomic structures depicted in abdominal and pelvic CT scans of young adults. Radiomics data have been extracted from contrast-enhanced and non-contrast-enhanced images in a reproducible manner, aiming to cover the current gap in open access radiomics datasets providing data that can be used for the development and validation of image-based machine learning models.

## Materials and Methods

### Patient Population

Data collection was performed with the approval of the Research Ethics Committee of our University hospital (683/20–1-2023) with a waiver for consent due to the anonymized retrospective nature of the study. The dataset population consisted of 531 patients who underwent an abdominal/pelvic CT examination between 2018 and 2023. Young adults aged ≥ 17 and ≤ 36 years old without any previously known disease were considered in this retrospective study and their abdominal and pelvic contrast and non-contrast enhanced CT scans were extracted from the hospital’s picture archiving and communications system (PACS). CT examinations were performed in GE or Siemens 64 slice scanners, with the following imaging parameters: beam collimation: 40 mm; field of view: 500 × 500 mm; matrix: 512 × 512; large body filter; rotation time: 0.4 s; tube voltage 120 kV; reconstruction slice thickness: 3.75 mm. Iodinated contrast (370 mg I/mL) was intravenously injected at a volume of 1 mL/kg of body weight, at an injection rate of 4 mL/s, and portal venous phase images were captured at 70 s following intravenous contrast injection. The manuscript has been written according to the STROBE checklist [12].

## Organ and Anatomical Structure Segmentation

Segmentation masks of various organs and anatomical structures were generated in an automatic manner, using the deep learning segmentation model TotalSegmentator [13, 14] which was accessed as an integrated tool in 3D Slicer (5.2.1 version) (slicer.org), a free and open-source platform for medical imaging data, to ensure reproducibility of segmentation results. Segmentations performed with TotalSegmentator were visually assessed by a senior radiology resident to ensure correct delineation of anatomical structures.

Radiomics features were extracted from each organ/anatomic structure detected by TotalSegmentator, using the open-source Python package PyRadiomics integrated into the 3D Slicer platform. The following feature classes were extracted: first order, gray level co-occurrence matrix (glcm), gray level dependence matrix (gldm), gray level run length matrix (glrlm), gray level size zone matrix (glszm), neighboring gray tone difference matrix (ngtdm), shape, shape2D, Laplacian of Gaussian (LoG), and wavelet-based features. A uniform bin width of 25 HU and voxel size resampling to 4 × 4 × 4 mm^3^ was performed to harmonize data extraction. LoG kernel size was set to 5 (Supplementary file 1). A bin width of 25 HU yields a minimum of 8 bins per examined structure. In addition, a voxel size of 4 × 4 × 4 mm^3^ will allow the use of our dataset not only with standard CT data but also with PET/CT data where the standard reconstruction voxel is 4 × 4 × 4 mm^3^.

Organs and anatomic structures of the reproductive system not detected by TotalSegmentator have not been included. Segmentation included abdominal organs (the liver, spleen, pancreas, adrenals, kidneys, gallbladder), muscles (paraspinal muscles, gluteal muscles, iliopsoas muscles), bones (lower ribs included in abdominal images, lower thoracic vertebrae, lumbar vertebrae, pelvic bones, and proximal femurs), and vessels (aorta and common iliac arteries, portal vein, inferior vena cava, and common iliac veins).

## Data Collection and Extraction—Exclusions

Imaging was performed for various indications including trauma and abdominal pain, to individuals without any previously known disease. In each examination, organs/structures that were identified as abnormal in radiology reports or identified as abnormal by the two experts that went through the dataset were excluded from radiomics feature extraction. Organs and anatomical structures were excluded for visible abnormal imaging findings such as traumatic lesions, organomegaly, identifiable focal lesions, the presence of inserted catheters, and the presence of kidney stones and fractured bones. All examinations were evaluated by at least two radiologists and only structures with a normal imaging appearance were included in the dataset. Every examination went through three stages of checks to verify to a possible extent that the cases were normal. First the original report was scoured by a senior radiology resident and a research fellow to extract all abnormalities noted. Each examination was co-reported by a senior radiology resident and an attending radiologist (minimum 5 years of experience as an attending). All images were also comprehensively evaluated to assess for any visible abnormality by a senior resident and an attending (40 years of experience) and finally available medical records were checked to identify any disease that could be related to abdominal pathology. All anatomical structures related to the gastrointestinal tract segmented by TotalSegmentator (colon, stomach, duodenum, small bowel) as well as the urinary bladder were excluded due to the variable imaging appearance related to the mobility, content and degree of distention which does not allow the extraction of reproducible radiomics features. All radiomics data can be accessed at [https://github.com/eliskape/Radiomics-Atlas].

## Results

### Dataset Characteristics

**Fig. 1:**
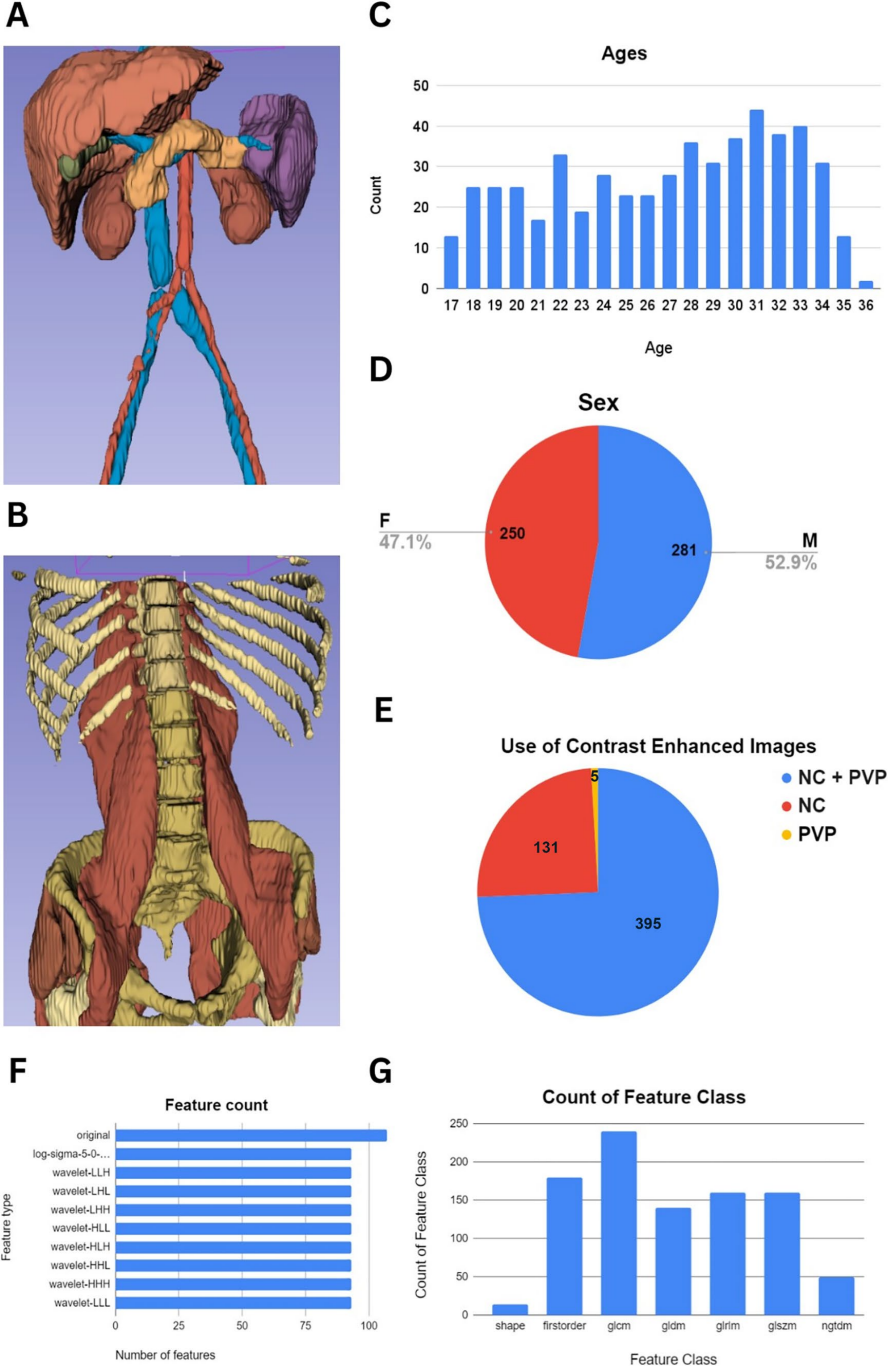
Dataset structure and content. **A** 3D illustration of organs and vessels segmented by TotalSegmentator.** B** 3D illustration of bone and muscle structures segmented by TotalSegmentator. **C** Age distribution of 531 patients. **D** Distribution of participant sex. **E** CT phases included in the dataset; NC + PV = 395, PV = 5, NC = 131. **F** Count of original, Laplacian of Gaussian, and wavelet-transformation features for each structure.** G** Count of Feature classes in the radiomics feature extraction for each patient. NC, no contrast; PVP, portal venous phase

**Fig. 2:**
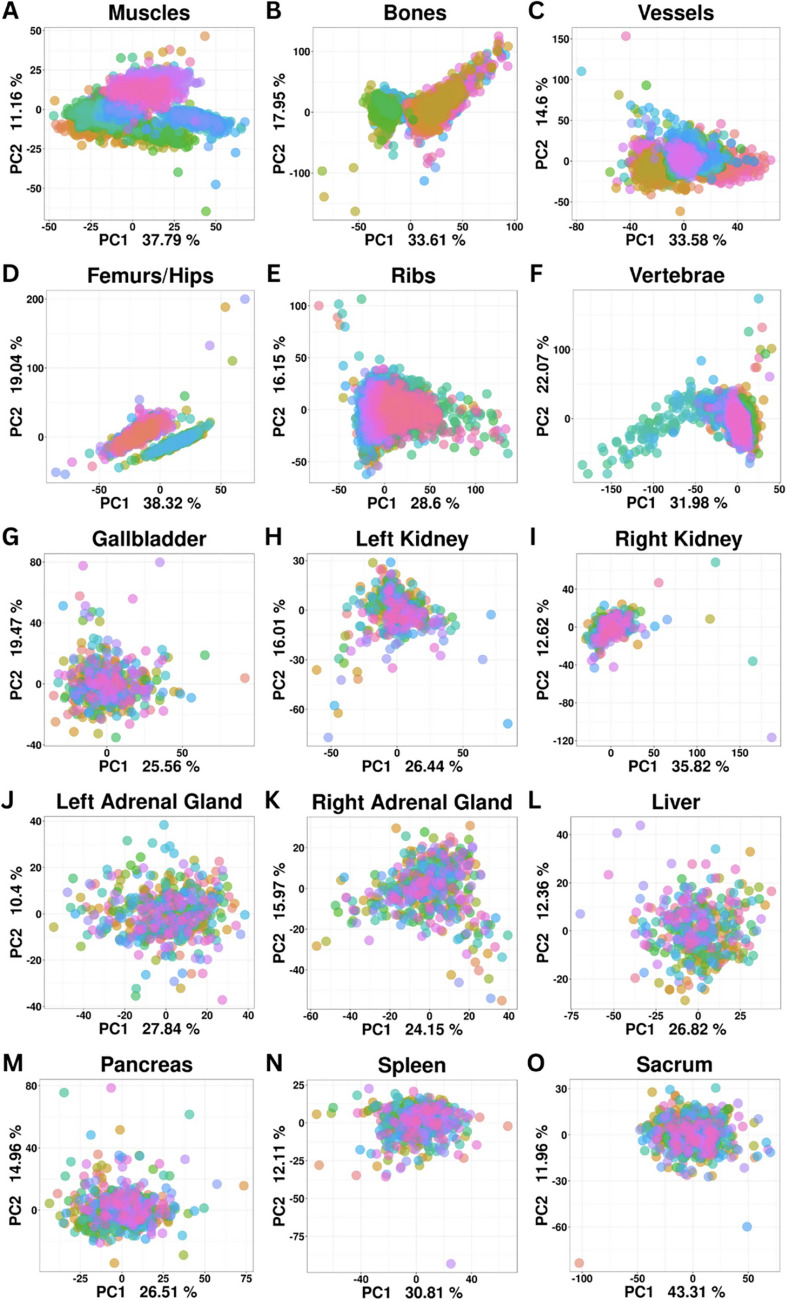
Principal component analysis (PCA) of the main groups of anatomical structures and organs detected by TotalSegmentator from non-contrast-enhanced CT scans. Each participant is depicted as a graph point with a different color

**Fig. 3:**
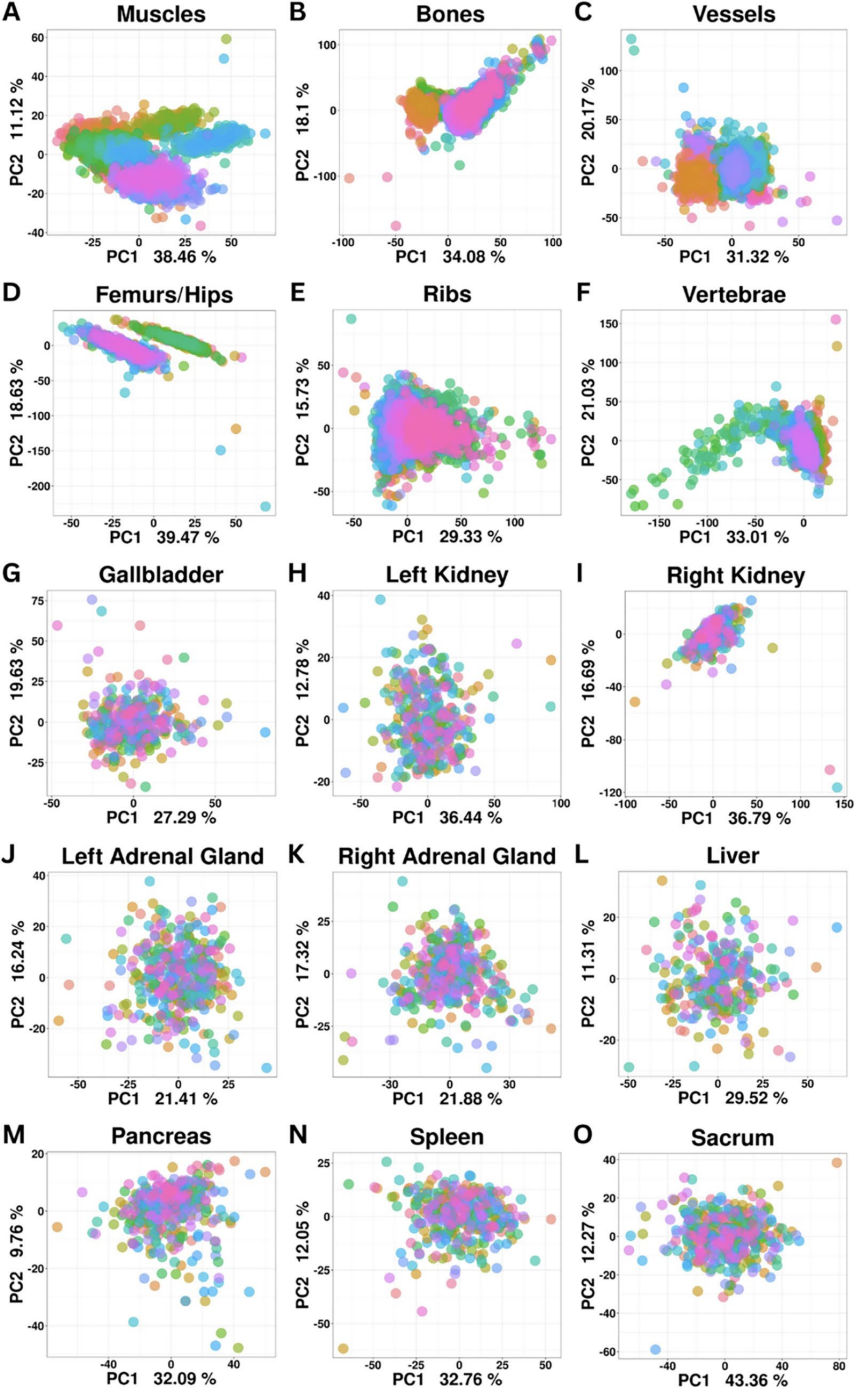
PCA of the main groups of anatomical structures and organs detected by TotalSegmentator from contrast-enhanced CT scans. Each participant is depicted as a graph point with a different color

Patient information and type of imaging in the RADAPT dataset are summarized in Table 1. A total of 531 unique patients were included in this dataset with a mean age of 26.8 ± 5.19 years old. The distribution of patient sexes was almost equally representative, including 281 males (52.9%) and 250 females (47.1%). A total number of 526 patients had non-contrast-enhanced images and 400 had portal venous phase contrast-enhanced imaging. More specifically, 5 patients had only portal venous phase contrast-enhanced imaging, 131 had only non-contrast-enhanced series, and 395 had both portal venous phase contrast-enhanced and non-contrast-enhanced series. The total number of non-contrast-enhanced series was 526, and the total number of portal venous phase contrast-enhanced series included in the study was 400. Parenchymal organs, muscles, vessels, and bones were included in the dataset. Principal component analysis (PCA) of the data demonstrated the homogeneous distribution of the samples without any major outliers. The RADAPT dataset structure and types of radiomics features extracted are depicted in Fig. 1. The total number of organs and anatomic structures included in this dataset are described in Table 2. Interestingly enough, despite the changes in radiomics information in contrast-enhanced images, the general distribution of radiomics data for individual organs did not exhibit outstanding differences between non-enhanced and contrast-enhanced scans, a finding consistent with the lack of localized lesions in target organs as expected in a healthy dataset (Figs. 2 and 3).

**Table 1:** Descriptive statistics of the RADAPT dataset

Data	Value
Number of patients	531
Mean age	26.8 ± 5.19
Sex	
Females	250
Males	281
Number of NC series	526
Number of PVP series	400
Maximum number of anatomical structures examined	53

**Table 2:** Total count of organs and anatomic structures segmented in the RADAPT dataset

			**Non-contrast**	**Contrast**
**Category**		**Organ**	Number of organs/anatomical structures	
**Abdominal organs**		Spleen	463	347
		Liver	384	272
		Pancreas	514	389
		Left kidney	442	348
		Right kidney	452	349
		Left adrenal gland	493	384
		Right adrenal gland	517	394
		Gallbladder	477	361
**Muscles**		Left erector spinae muscle	525	397
		Right erector spinae muscle	525	397
		Left gluteus maximus	523	397
		Right gluteus maximus	523	397
		Left gluteus medius	523	397
		Right gluteus medius	523	397
		Left gluteus minimus	523	397
		Right gluteus minimus	523	397
		Left iliopsoas muscle	525	397
		Right iliopsoas muscle	525	397
**Bones**		L1 vertebra	515	391
		L2 vertebra	516	390
		L3 vertebra	516	391
		L4 vertebra	513	387
		L5 vertebra	516	390
		Left rib 6	509	384
		Left rib 7	521	392
		Left rib 8	522	395
		Left rib 9	523	397
		Left rib 10	524	397
		Left rib 11	524	397
		Left rib 12	520	392
		Right rib 6	504	379
		Right rib 7	520	391
		Right rib 8	522	394
		Right rib 9	523	397
		Right rib 10	524	397
		Right rib 11	525	397
		Right rib 12	522	394
		T9 vertebra	486	367
		T10 vertebra	511	388
		T11 vertebra	518	391
		T12 vertebra	519	394
		Left hip	519	393
		Right hip	520	393
		Left femur	512	388
		Right femur	515	390
		Sacrum	516	389
**Vessels**	Arteries	Aorta	523	396
		Left common iliac artery	525	397
		Right common iliac artery	525	397
	Veins	Portal vein	492	397
		Inferior vena cava	525	397
		Left common iliac vein	525	397
		Right common iliac vein	525	397

## Dataset Size and Structure

Radiomics data of the RADAPT dataset have been extracted from a total of 53 structures. The radiomics features extracted from each patient were stored in a GitHub repository [ https://github.com/eliskape/Radiomics-Atlas] in Excel files, divided by organ/anatomic structure, as listed in Table 2. The organs and anatomic structures with visible imaging findings have been removed and each ID number corresponds to one unique patient.

## Discussion

We describe the RADAPT dataset, a radiomics dataset of 53 major healthy anatomical structures from abdominal and pelvic CT scans derived from 531 young adults aged ≥ 17 and ≤ 36 years old. This open access dataset can be used for the development and validation of radiomics-based machine learning models.

While molecular characterization atlases based on omics technologies showed rapid growth [1–6], the scarcity of open access radiomics datasets, especially those encompassing multiple normal organs, remains a significant limitation for validating new machine learning models. This dataset, which includes 53 organs/anatomical structures from the abdominal and pelvic area, provides a valuable tool for researchers looking to develop, test, and improve machine learning models in the field of radiology and image-based omics extraction. The lack of normal control groups is a major problem in research manuscripts [15], especially when omics analyses are employed. Our dataset enables off-the-shelf comparison of radiomics data to a diverse healthy population to enable the comparison of pathological to normal tissues. In addition, radiomics has been largely integrated with other omics datasets to extract associations linking the biological with the imaging phenotype of certain diseases [16–18]. Our dataset can be potentially integrated with other atlases [6] of healthy omics (epigenomics [19], transcriptomics [1], proteomics [5], metabolomics [2]) to derive links between biological and imaging cues.

All examinations were performed using common imaging parameters used by the majority of scanners and departments worldwide. The examination protocol may change according to the disease imaged; however, this is rather related to the contrast-enhanced phases that are acquired (e.g., some exams may contain arterial phase images or delayed contrast-enhanced images depending on the disease imaged). However, the majority if not all abdominal scans performed worldwide would have at least one or both of (i) non-enhanced or (ii) portal venous phase images. This is the reason that these types of images were selected in our study, to match the majority of examinations. Common acquisition parameters (e.g., tube voltage, beam collimation) were also used to increase the comparability of the data. Potential uses of our dataset include but are not limited to (a) integration with other omics datasets, (b) supplementation of external validation sets for the evaluation of machine learning algorithms, and (c) utilization as a control group independently or together with other normal data for studies examining a certain disease and others.

There are some inherent limitations in this study. The dataset is derived from a specific age group of young adults limited to ≥ 17 and ≤ 36 years old, which may not be entirely representative of the broader population. Age-related changes in organ morphology and tissue characteristics might introduce variability when applying this radiomics reference atlas to older populations. Nonetheless, this specific age range was chosen by design with the goal of representing healthy tissues and organs. Another limitation is that there is a possibility that an undiagnosed/undocumented underlying disease could be present in some of our patients. Nonetheless, a comprehensive analysis of all available patient data was done to ensure that no relevant disease was on record and that no visible abnormal imaging finding was included. We believe that this radiomics atlas of abdominal and pelvic CT scans is offering a major step towards bridging the gap in open access radiomics datasets and setting the stage for more comprehensive studies in the field of radiology and artificial intelligence.

### Supplementary Information

Below is the link to the electronic supplementary material.

Supplementary file1 (DOCX 14 KB)

## Data Availability

All data used in this manuscript can be found at https://github.com/eliskape/Radiomics-Atlas.
