# Supplementary material for: Developing a Radiomics Atlas Dataset of normal Abdominal and Pelvic computed Tomography (RADAPT)
Source: J Imaging Inform Med. 2024 Feb 21;37(4):1273–81. doi: 10.1007/s10278-024-01028-7 (PMC11300734; doi:10.1007/s10278-024-01028-7)
Supplement: Supplementary file 1 — Supplementary file1 (DOCX 14 KB) [file 10278_2024_1028_MOESM1_ESM.docx]

*Feature classes:*

- *first order*
- *gray level co-occurence matrix (glcm)*
- *gray level dependence matrix (gldm)*
- *gray level run length matrix (glrlm)*
- *gray level size zone matrix (glszm)*
- *neighbouring gray tone difference matrix (ngtdm)*
- *shape*
- *shape2D*
- *laplacian of gaussian (LoG)*
- *wavelet-transformed features*

*Bin width = 25.00*

*Voxel size resampling = 4x4x4 mm^3^*

*LoG kernel size was set to 5.*

*Enforce symmetrical GLCM = TRUE*
